# Supplementary material for: The association between number and ages of children and the physical activity of mothers: Cross-sectional analyses from the Southampton Women’s Survey
Source: PLoS One. 2022 Nov 16;17(11):e0276964. doi: 10.1371/journal.pone.0276964 (PMC9668156; doi:10.1371/journal.pone.0276964)
Supplement: S1 Appendix — (DOCX) [file pone.0276964.s001.docx]

**STROBE^a^ statement – checklist of items that should be included in reports of cross-sectional studies for “****The association between number and ages of children and the physical activity of mothers: cross-sectional analyses from the Southampton Women’s Survey”**

|  | **Item no** | **Recommendation** | **Page number in manuscript** |
| --- | --- | --- | --- |
| **Title and abstract** | 1 | 1. Indicate the study’s design with a commonly used term in the title or the abstract | 1 |
|  |  | 1. Provide in the abstract an informative and balanced summary of what was done and what was found | 2-3 |
| **Introduction** | 2 | Explain the scientific background and rationale for the investigation being reported | 4-5 |
| Background/rationale | 3 | State specific objectives, including any prespecified hypotheses | 5 |
| **Methods** |  |  |  |
| Study design | 4 | Present key elements of study design early in the paper | 5-11 |
| Setting | 5 | Describe the setting, locations, and relevant dates, including periods of recruitment, exposure, follow-up, and data collection | 5-6 |
| Participants | 6 | (*a*) Give the eligibility criteria, and the sources and methods of selection of participants | 5-6 |
| Variables | 7 | Clearly define all outcomes, exposures, predictors, potential confounders, and effect modifiers. Give diagnostic criteria, if applicable | 6-9 |
| Data sources/ measurement | 8 | For each variable of interest, give sources of data and details of methods of assessment (measurement). Describe comparability of assessment methods if there is more than one group | 6-9 |
| Bias | 9 | Describe any efforts to address potential sources of bias | 6 |
| Study size | 10 | Explain how the study size was arrived at | 11, Figure 1 |
| Quantitative variables | 11 | Explain how quantitative variables were handled in the analyses. If applicable, describe which groupings were chosen and why | 6-9 |
| Statistical methods | 12 | (*a*) Describe all statistical methods, including those used to control for confounding | 9-11 |
|  |  | (*b*) Describe any methods used to examine subgroups and interactions | 10-11 |
|  |  | (*c*) Explain how missing data were addressed | 8-11 |
|  |  | (*d*) If applicable, describe analytical methods taking account of sampling strategy | 10 |
|  |  | (*e*) Describe any sensitivity analyses | 11 |
| **Results** |  |  |  |
| Participants | 13 | (a) Report numbers of individuals at each stage of study—eg numbers potentially eligible, examined for eligibility, confirmed eligible, included in the study, completing follow-up, and analysed | 11, Figure 1 |
|  |  | (b) Give reasons for non-participation at each stage | Figure 1 |
|  |  | (c) Consider use of a flow diagram | Figure 1 |
| Descriptive data | 14 | (a) Give characteristics of study participants (eg demographic, clinical, social) and information on exposures and potential confounders | Tables 2 and 3 |
|  |  | (b) Indicate number of participants with missing data for each variable of interest | Tables 2 and 3 |
| Outcome data | 15 | Report numbers of outcome events or summary measures | Tables 2 and 3 |
| Main results | 16 | (*a*) Give unadjusted estimates and, if applicable, confounder-adjusted estimates and their precision (eg, 95% confidence interval). Make clear which confounders were adjusted for and why they were included | 14-17; S2 and S3 Appendices |
|  |  | (*b*) Report category boundaries when continuous variables were categorized | Tables 2 and 3 |
|  |  | (*c*) If relevant, consider translating estimates of relative risk into absolute risk for a meaningful time period | n/a |
|  | 17 | Report other analyses done—eg analyses of subgroups and interactions, and sensitivity analyses | 14, 17, S8 and S9 Appendices |
| **Discussion** |  |  |  |
| Key results | 18 | Summarise key results with reference to study objectives | 18 |
| Limitations | 19 | Discuss limitations of the study, taking into account sources of potential bias or imprecision. Discuss both direction and magnitude of any potential bias | 20-21 |
| Interpretation | 20 | Give a cautious overall interpretation of results considering objectives, limitations, multiplicity of analyses, results from similar studies, and other relevant evidence | 18-22 |
| Generalisability | 21 | Discuss the generalisability (external validity) of the study results | 21-22 |
| **Other information** |  |  |  |
| Funding | 22 | Give the source of funding and the role of the funders for the present study and, if applicable, for the original study on which the present article is based | To be inserted into manuscript by journal |

^a^von Elm E, Altman DG, Egger MJ, Pocock SJ, Gotzsche PC, Vandenbroucke JP. Strengthening the Reporting of Observational Studies in Epidemiology (STROBE) Statement: guidelines for reporting observational studies. Ann Intern Med. 2007;147(8):573-7.
