# Supplementary material for: The association between number and ages of children and the physical activity of mothers: Cross-sectional analyses from the Southampton Women’s Survey
Source: PLoS One. 2022 Nov 16;17(11):e0276964. doi: 10.1371/journal.pone.0276964 (PMC9668156; doi:10.1371/journal.pone.0276964)
Supplement: S3 Appendix — (PDF) [file pone.0276964.s003.pdf]

Direct acyclic graph for analyses investigating the association between ages of children and maternal physical activity

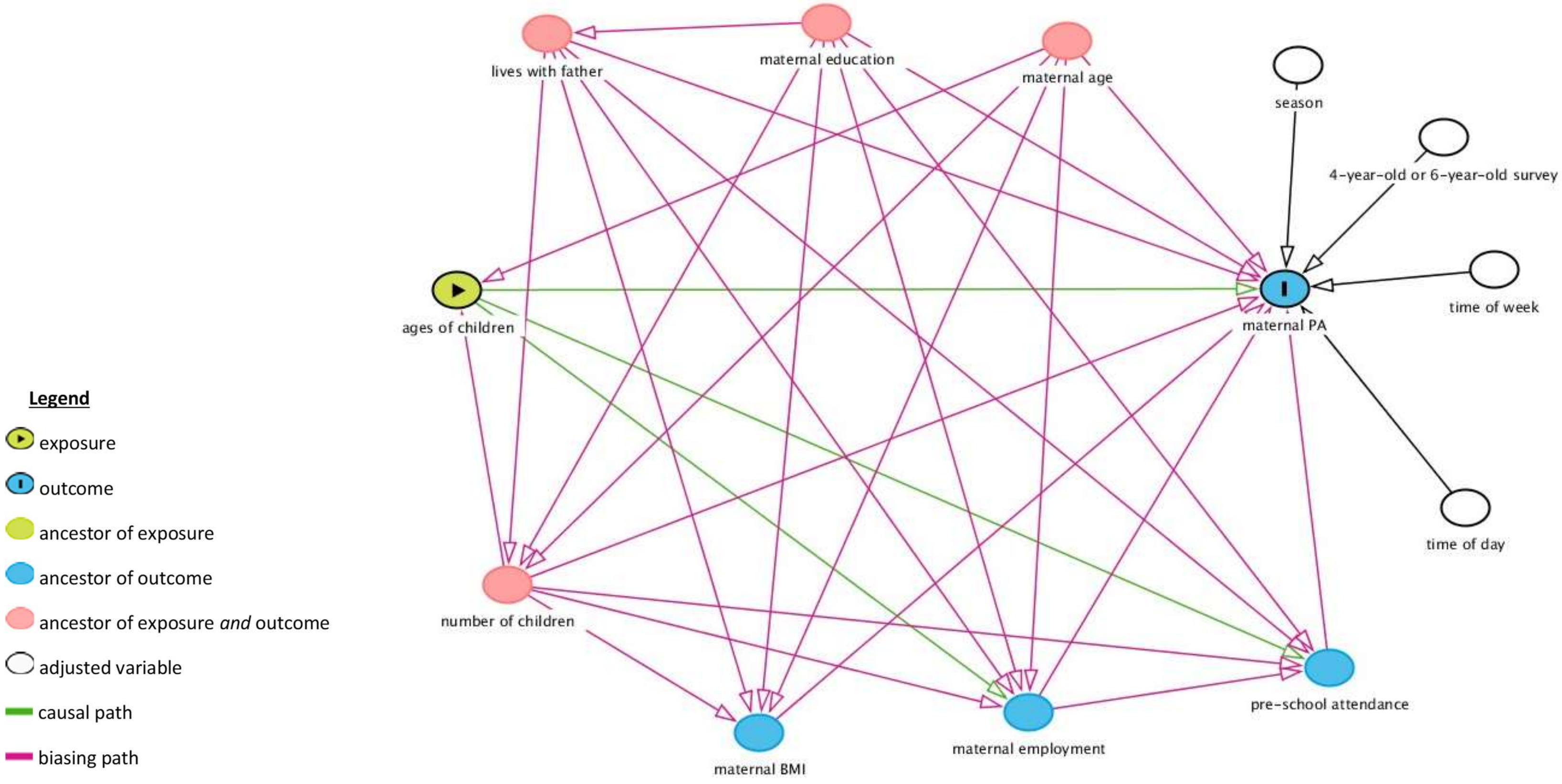

Diagram created using Dagitty (Textor et al. 2016)

Confounders adjusted for: maternal age; number of children. Competing exposures adjusted for: age 4y or age 6y survey; time of week; season.
