# Supplementary material for: The association between number and ages of children and the physical activity of mothers: Cross-sectional analyses from the Southampton Women’s Survey
Source: PLoS One. 2022 Nov 16;17(11):e0276964. doi: 10.1371/journal.pone.0276964 (PMC9668156; doi:10.1371/journal.pone.0276964)
Supplement: S4 Appendix — (DOCX) [file pone.0276964.s004.docx]

**A** **Comparison of descriptive characteristics from initial survey or at time of birth of index child between women included in analyses and those women without accelerometer data (n=2972)**

|  | **Included**  **(n=848)** | **Not included**  **(n=2124)** | **p-value for difference in values between groups^a^** |
| --- | --- | --- | --- |
| **Age of mother at birth of index child, years (mean, SD)** | 30.9 (3.6) | 30.6 (3.9) | 0.06 |
| **BMI at initial survey, kg/m^2^ (median, IQR)** | 24.1 (21.9-27.2) | 24.1 (21.8-27.4) | 0.70 |
| **Qualification level from initial survey^b^ (%, n)**  **None**  **CSE**  **O-levels**  **A-levels**  **HND**  **Degree** | 1.4 (12)  9.2 (78)  28.0 (236)  31.4 (265)  6.9 (58)  23.1 (195) | 3.9 (83)  9.5 (202)  29.5 (625)  29.3 (621)  6.0 (127)  21.8 (461) | 0.01 |

^a^This was calculated from a t-test for age at birth of index child, non-parametric equality of medians for BMI, chi-squared tests for education levels. ^b^From lowest to highest level of education in 6 categories.

Missing values: <1% (n<10) for highest qualification level and age of mother at birth of index child, <1% (n=25) for BMI. BMI=Body Mass Index; CSE=certificate of secondary education; HND=higher national diploma; IQR=interquartile range; SD=standard deviation.
