# Supplementary material for: The association between number and ages of children and the physical activity of mothers: Cross-sectional analyses from the Southampton Women’s Survey
Source: PLoS One. 2022 Nov 16;17(11):e0276964. doi: 10.1371/journal.pone.0276964 (PMC9668156; doi:10.1371/journal.pone.0276964)
Supplement: S5 Appendix — (DOCX) [file pone.0276964.s005.docx]

**Stratified associations between ages and number of children and maternal LMVPA**

**Table S5.1. Associations between ages of children and maternal LMVPA by number of children**

|  | **Beta [95%CI] for LMVPA (mins per day)** | | | | | |
| --- | --- | --- | --- | --- | --- | --- |
|  | **All days** | | **All weekdays** | | **All weekend days** | |
| **1 child**  **(ref: younger children)** |  | |  | |  | |
| **School-aged** | -0.3 [-22.9, 22.3] | p=0.98 | 8.8 [-15.3, 33.0] | p=0.47 | -21.6 [-56.2, 13.0] | p=0.22 |
|  |  | |  | |  | |
| **2 children**  **(ref: younger children)** |  | |  | |  | |
| **School-aged** | -34.0 [-64.1, -3.9] | p=0.03 | -32.2 [-64.3, -0.2] | p=0.05 | -47.0 [-83.9, -10.2] | p=0.01 |
| **Both age groups** | -10.0 [-28.5, 8.6] | p=0.29 | -7.2 [-27.4, 12.9] | p=0.48 | -32.1 [-56.5, -7.6] | p=0.01 |
|  |  | |  | |  | |
| **>3 children (ref: younger children)** |  | |  | |  | |
| **School-aged** | -108.0 [-168.0, -48.1] | p<0.01 | -80.2 [-142.8, -17.6] | p=0.01 | -120.5 [-201.1, -39.8] | p<0.01 |
| **Both age groups** | -81.2 [-129.3, -33.0] | p<0.01 | -54.0 [-104.4, -3.6] | p=0.04 | -86.2 [-152.7, -19.8] | p=0.01 |

Models adjusted for age of mother, season, age 4y or age 6y survey, time of week (for all days analyses). LMVPA=light, moderate or vigorous physical activity; 95%CI=95% confidence interval.

**Table S5.2. Associations between number of children and maternal LMVPA by ages of children**

|  | **Beta [95%CI] for LMVPA (mins per day)** | | | | | |
| --- | --- | --- | --- | --- | --- | --- |
|  | **All days** | | **All weekdays** | | **All weekend days** | |
| **Younger children**  **(ref: 1 child)** |  | |  | |  | |
| **2 children** | 42.6 [16.4, 68.8] | p<0.01 | 45.4 [18.0, 72.8] | p<0.01 | 33.6 [2.3, 65.0] | p=0.04 |
| **>3 children** | 49.9 [-6.1, 105.9] | p=0.08 | 55.6 [-3.0, 114.2] | p=0.06 | 34.5 [-30.0, 99.0] | p=0.30 |
|  |  | |  | |  | |
| **School-aged**  **(ref: 1 child)** |  | |  | |  | |
| **2 children** | 13.4 [-13.0, 39.8] | p=0.32 | 11.3 [-16.0, 38.7] | p=0.42 | 21.0 [-11.5, 53.5] | p=0.21 |
| **>3 children** | 13.8 [-19.9, 47.4] | p=0.42 | 13.9 [-20.8, 48.6] | p=0.43 | 16.2 [-25.0, 57.4] | p=0.44 |
|  |  | |  | |  | |
| **Both age groups**  **(ref: 2 children)** |  | |  | |  | |
| **>3 children** | 28.4 [10.7, 46.2] | p<0.01 | 28.0 [9.3, 46.8] | p<0.01 | 19.2 [-3.2, 41.7] | p=0.09 |

Models adjusted for age of mother, maternal highest qualification level, living with father, season, age 4y or age 6y survey, time of week (for all days analyses). LMVPA=light, moderate or vigorous physical activity; 95%CI=95% confidence interval.
