# Supplementary material for: The association between number and ages of children and the physical activity of mothers: Cross-sectional analyses from the Southampton Women’s Survey
Source: PLoS One. 2022 Nov 16;17(11):e0276964. doi: 10.1371/journal.pone.0276964 (PMC9668156; doi:10.1371/journal.pone.0276964)
Supplement: S6 Appendix — (DOCX) [file pone.0276964.s006.docx]

**Associations between ages and number of children and maternal MVPA levels by time of the day**

|  | **Percentage difference [95%CI] in MVPA^a^** | | | | | |
| --- | --- | --- | --- | --- | --- | --- |
|  | **Weekday morning**  **(6-9am)** | **Weekday School/ work day**  **(9am-3pm)** | **Weekday Late afternoon**  **(3pm-7pm)** | **Weekday Evening**  **(7pm-11pm)** | **Weekend Day**  **(6am-7pm)** | **Weekend evening (7pm-11pm)** |
| **Ages of children**  **(ref: younger children)** |  |  |  |  |  |  |
| **School-aged** | 81.1 [49.7, 119.1] | 38.1 [11.7, 70.8] | 79.6 [47.1, 119.3] | 11.7 [-2.8, 28.3] | 32.6 [-1.6, 78.7] | 27.4 [8.0, 50.2] |
| **Both age groups** | 65.3 [44.4, 89.2] | 26.3 [8.3, 47.2] | 48.2 [28.3, 71.1] | 8.9 [-1.8, 20.7] | 19.6 [-4.2, 49.5] | 20.1 [5.9, 36.2] |
|  |  |  |  |  |  |  |
| **Number of children**  **(ref: 1 child)** |  |  |  |  |  |  |
| **2 children** | -15.1 [-26.6, -1.8] | -0.2 [-14.9, 17.1] | -10.2 [-22.7, 4.3] | -7.1 [-15.9, 2.7] | -15.3 [-31.7, 5.0] | 2.4 [-9.1, 15.3] |
| **>3 children** | -18.8 [-31.1, -4.3] | 1.5 [-15.1, 21.5] | -8.8 [-23.0, 8.0] | 2.7 [-8.2, 14.9] | -23.3 [-39.6, -2.5] | 0.5 [-11.9, 14.7] |

^a^Percentage difference in MVPA is calculated from the geometric mean ratio as MVPA was log-transformed for analyses.

Ages of children models adjusted for age of mother, number of children, season, age 4y or age 6y survey. Number of children models adjusted for age of mother, maternal highest qualification level, living with father, season, age 4y or age 6y survey. 835 mothers were included in weekday and 738 in weekend analyses related to the ages of children. 831 mothers were included in weekday and 734 in weekend analyses related to the number of children. MVPA=moderate or vigorous physical activity; 95%CI=95% confidence interval.
