# Supplementary material for: The association between number and ages of children and the physical activity of mothers: Cross-sectional analyses from the Southampton Women’s Survey
Source: PLoS One. 2022 Nov 16;17(11):e0276964. doi: 10.1371/journal.pone.0276964 (PMC9668156; doi:10.1371/journal.pone.0276964)
Supplement: S7 Appendix — (DOCX) [file pone.0276964.s007.docx]

**Stratified associations between ages and number of children and maternal LMVPA levels by time of the day**

**Table S7.1. Associations between ages of children and maternal LMVPA levels by number of children by time of the day**

|  | **Beta Coefficients [95%CI] for LMVPA (mins per day)** | | | | | |
| --- | --- | --- | --- | --- | --- | --- |
|  | **Weekday morning**  **(6-9am)** | **Weekday School/ work day**  **(9am-3pm)** | **Weekday Late afternoon**  **(3pm-7pm)** | **Weekday Evening**  **(7pm-11pm)** | **Weekend Day**  **(6am-7pm)** | **Weekend evening (7pm-11pm)** |
| **1 child**  **(ref: younger children)** |  |  |  |  |  |  |
| **School-aged** | 9.5 [2.9, 16.2] | -5.1 [-19.7, 9.5] | 10.8 [2.1, 19.5] | 4.1 [-3.9, 12.0] | -13.1 [-44.8, 18.6] | 6.3 [-3.3, 15.8] |
| **2 children**  **(ref: younger children)** |  |  |  |  |  |  |
| **School-aged** | 0.9 [-7.3, 9.2] | -17.9 [-35.6, -0.3] | -9.6 [-19.8, 0.7] | -1.6 [-10.8, 7.5] | -49.8 [-82.7, -16.9] | 1.5 [-8.9, 11.8] |
| **Both age categories** | 5.4 [0.2, 10.5] | -8.6 [-19.9, 2.7] | -3.6 [-10.2, 3.0] | -0.6 [-6.5, 5.3] | -35.0 [-56.6, -13.4] | 1.5 [-5.5, 8.5] |
| **>3 children**  **(ref: younger children)** |  |  |  |  |  |  |
| **School-aged** | -6.1 [-23.6, 11.4] | -40.9 [-75.8, -6.1] | -20.5 [-40.5, -0.5] | -20.9 [-40.3, -1.5] | -109.7 [-182.0, -37.4] | -2.1 [-26.9, 22.6] |
| **Both age categories** | -6.3 [-20.4, 7.8] | -25.0 [-53.2, 3.2] | -10.6 [-26.8, 5.7] | -15.1 [-31.0, 0.8] | -74.7 [-135.6, -13.9] | 3.6 [-17.3, 24.4] |

Models adjusted for age of mother, season, age 4y or age 6y survey. Analyses are for 835 mothers for weekdays and 738 for weekends. LMVPA=light, moderate or vigorous physical activity; 95% CI=95% confidence interval.

**Table S7.2. Associations between number of children and maternal LMVPA levels by ages of children by time of the day**

|  | **Beta Coefficients [95% CI] for LMVPA (mins per day)** | | | | | |
| --- | --- | --- | --- | --- | --- | --- |
|  | **Weekday morning**  **(6-9am)** | **Weekday School/ work day**  **(9am-3pm)** | **Weekday Late afternoon**  **(3pm-7pm)** | **Weekday Evening**  **(7pm-11pm)** | **Weekend Day**  **(6am-7pm)** | **Weekend evening (7pm-11pm)** |
| **Younger children**  **(ref: 1 child)** |  |  |  |  |  |  |
| **2 children** | 3.3 [-4.0, 10.7] ] | 29.8 [14.6, 45.1] | 13.5 [4.5, 22.6] | 5.9 [-2.1, 13.9] | 26.8 [-0.9, 54.4] | 3.9 [-4.7, 12.5] |
| **>3 children** | 4.0 [-11.7, 19.8] | 39.5 [7.1, 72.0] | 16.1 [-3.2, 35.4] | 7.9 [-9.2, 24.9] | 43.1 [-15.9, 102.0] | 1.4 [-17.0, 19.8] |
| **School-aged**  **(ref: 1 child)** |  |  |  |  |  |  |
| **2 children** | 4.6[-2.7, 11.8] | 11.1 [-4.3, 26.5] | -2.4 [-11.4, 6.6] | -0.9 [-8.4, 6.7] | 12.2 [-16.7, 41.1] | -0.5 [-9.8, 8.7] |
| **>3 children** | 3.9 [-5.3, 13.1] | 16.2 [-3.3, 35.8] | 1.0 [-10.4, 12.4] | 3.6 [-6.0, 13.1] | 9.3 [-27.2, 45.8] | 11.5 [-0.2, 23.1] |
| **Both age groups**  **(ref: 2 children)** |  |  |  |  |  |  |
| **>3 children** | 0.1 [-4.8, 5.0] | 17.1 [6.9, 27.4] | 6.1 [0.3, 11.8] | 4.4 [-1.1, 9.9] | 15.9 [-4.2, 36.0] | 8.0 [1.5, 14.5] |

Models adjusted for age of mother, maternal highest qualification level, living with father, season, age 4y or age 6y survey. Analyses are for 831 mothers for weekdays and 734 for weekends. LMVPA=light, moderate or vigorous physical activity; 95% CI=95% confidence interval.
