# Supplementary material for: The association between number and ages of children and the physical activity of mothers: Cross-sectional analyses from the Southampton Women’s Survey
Source: PLoS One. 2022 Nov 16;17(11):e0276964. doi: 10.1371/journal.pone.0276964 (PMC9668156; doi:10.1371/journal.pone.0276964)
Supplement: S8 Appendix — (DOCX) [file pone.0276964.s008.docx]

**Sensitivity analyses for MVPA**

**Table S8.1. Associations between ages and number of children and maternal MVPA levels for the subset of mothers with 3 or more valid days of accelerometer data (all days available) (n=801)***

|  | **Percentage difference**  **[95%CI] in MVPA^a^** | |
| --- | --- | --- |
| **Ages of children**  **(ref: younger children)** |  | |
| **School-aged** | 46.8 [21.3, 77.5] | p<0.01 |
| **Both age groups** | 42.6 [24.8, 63.0] | p<0.01 |
|  |  | |
| **Number of children**  **(ref: 1 child)** |  | |
| **2 children** | -12.4 [-24.3, 1.6] | p=0.08 |
| **>3 children** | -11.7 [-25.2, 4.2] | p=0.14 |

*n=797 for number of children analysis. ^a^Percentage difference in MVPA is calculated from the geometric mean ratio as MVPA was log-transformed for analyses.

Ages of children model adjusted for age of mother, number of children, season, age 4y or age 6y survey, time of week. Number of children model adjusted for age of mother, maternal highest qualification level, living with father, season, age 4y or age 6y survey, time of week. MVPA=moderate or vigorous physical activity; 95%CI=95% confidence interval.

**Table S8.2. Associations between ages and number of children and maternal MVPA levels for the subset of mothers with 5 or more valid days of accelerometer data (all days available) (n=725)***

|  | **Percentage difference**  **[95%CI] in MVPA^a^** | |
| --- | --- | --- |
| **Ages of children**  **(ref: younger children)** |  | |
| **School-aged** | 57.3 [28.7, 92.3] | p<0.01 |
| **Both age groups** | 47.2 [28.0, 69.4] | p<0.01 |
|  |  | |
| **Number of children**  **(ref: 1 child)** |  | |
| **2 children** | -9.1 [-22.2, 6.3] | p=0.23 |
| **>3 children** | -7.6 [-22.3, 9.9] | p=0.37 |

*n=721 for number of children analysis. ^a^Percentage difference in MVPA is calculated from the geometric mean ratio as MVPA was log-transformed for analyses.

Ages of children model adjusted for age of mother, number of children, season, age 4y or age 6y survey, time of week. Number of children model adjusted for age of mother, maternal highest qualification level, living with father, season, age 4y or age 6y survey, time of week. MVPA=moderate or vigorous physical activity; 95%CI=95% confidence interval.

**Table S8.3. Associations between ages and number of children and maternal MVPA levels with those with missing data relating to number of younger or older children assumed to have none in that category (all days available) (n=1009)***

|  | **Percentage difference**  **[95%CI] in MVPA^a^** | |
| --- | --- | --- |
| **Ages of children**  **(ref: younger children)** |  | |
| **School-aged** | 47.8 [24.3, 75.8] | p<0.01 |
| **Both age groups** | 41.6 [25.3, 60.1] | p<0.01 |
|  |  | |
| **Number of children**  **(ref: 1 child)** |  | |
| **2 children** | -10.7 [-21.8, 2.0] | p=0.10 |
| **>3 children** | -14.5 [-26.6, -0.4] | p=0.04 |

*n=913 for number of children analysis. ^a^Percentage difference in MVPA is calculated from the geometric mean ratio as MVPA was log-transformed for analyses.

Ages of children model adjusted for age of mother, number of children, season, age 4y or age 6y survey, time of week. Number of children model adjusted for age of mother, maternal highest qualification level, living with father, season, age 4y or age 6y survey, time of week. MVPA=moderate or vigorous physical activity; 95%CI=95% confidence interval.
