# Supplementary material for: The association between number and ages of children and the physical activity of mothers: Cross-sectional analyses from the Southampton Women’s Survey
Source: PLoS One. 2022 Nov 16;17(11):e0276964. doi: 10.1371/journal.pone.0276964 (PMC9668156; doi:10.1371/journal.pone.0276964)
Supplement: S9 Appendix — (DOCX) [file pone.0276964.s009.docx]

**Sensitivity analyses for LMVPA**

**Table S9.1. Associations between ages of children and maternal LMVPA levels by number of children for the subset of mothers with 3 or more valid days of accelerometer data (all days available) (n=801)**

|  | **Beta [95%CI] for LMVPA**  **(mins per day)** | |
| --- | --- | --- |
| **1 child**  **(ref: younger children)** |  | |
| **School-aged** | 3.5 [-19.6, 26.7] | p=0.77 |
|  |  | |
| **2 children**  **(ref: younger children)** |  | |
| **School-aged** | -36.8 [-67.8, -5.9] | p=0.02 |
| **Both age groups** | -13.4 [-32.3, 5.5] | p=0.16 |
|  |  | |
| **>3 children**  **(ref: younger children)** |  | |
| **School-aged** | -115.4 [-176.3, -54.6] | p<0.01 |
| **Both age groups** | -87.1 [-136.2, -38.0] | p<0.01 |

Models adjusted for age of mother, season, age 4y or age 6y survey, time of week. LMVPA=light, moderate or vigorous physical activity; 95%CI=95% confidence interval.

**Table S9.2. Associations between number of children and maternal LMVPA levels by ages of children for the subset of mothers with 3 or more valid days of accelerometer data (all days available) (n=797)**

|  | **Beta [95%CI] for LMVPA**  **(mins per day)** | |
| --- | --- | --- |
| **Younger children**  **(ref: 1 child)** |  | |
| **2 children** | 43.7 [16.9, 70.5] | p<0.01 |
| **>3 children** | 49.7 [-6.6, 106.0] | p=0.08 |
|  |  | |
| **School-aged**  **(ref: 1 child)** |  | |
| **2 children** | 14.3 [-12.8, 41.4] | p=0.30 |
| **>3 children** | 14.1 [-20.2, 48.5] | p=0.42 |
|  |  | |
| **Both age groups**  **(ref: 2 children)** |  | |
| **>3 children** | 29.5 [11.2, 47.7] | p<0.01 |

Models adjusted for age of mother, maternal highest qualification level, living with father, season, age 4y or age 6y survey, time of week. LMVPA=light, moderate or vigorous physical activity; 95%CI=95% confidence interval.

**Table S9.3. Associations between ages of children and maternal LMVPA levels by number of children for the subset of mothers with 5 or more valid days of accelerometer data (all days available) (n=725)**

|  | **Beta [95%CI] for LMVPA**  **(mins per day)** | |
| --- | --- | --- |
| **1 child**  **(ref: younger children)** |  | |
| **School-aged** | 3.8 [-21.6, 29.2] | p=0.77 |
|  |  | |
| **2 children**  **(ref: younger children)** |  | |
| **School-aged** | -34.7 [-67.8, -1.7] | p=0.04 |
| **Both age groups** | -11.5 [-31.6, 8.5] | p=0.26 |
|  |  | |
| **>3 children**  **(ref: younger children)** |  | |
| **School-aged** | -107.7 [-172.5, -42.8] | p<0.01 |
| **Both age groups** | -80.7 [-134.0, -27.5] | p<0.01 |

Models adjusted for age of mother, season, age 4y or age 6y survey, time of week. LMVPA=light, moderate or vigorous physical activity; 95%CI=95% confidence interval.

**Table S9.4. Associations between number of children and maternal LMVPA levels by ages of children for the subset of mothers with 5 or more valid days of accelerometer data (all days available) (n=721)**

|  | **Beta [95%CI] for LMVPA**  **(mins per day)** | |
| --- | --- | --- |
| **Younger children**  **(ref: 1 child)** |  | |
| **2 children** | 41.7 [12.7, 70.7] | p=0.01 |
| **>3 children** | 48.9 [-10.9, 108.8] | p=0.11 |
|  |  | |
| **School-aged**  **(ref: 1 child)** |  | |
| **2 children** | 15.1 [-12.7, 42.9] | p=0.29 |
| **>3 children** | 16.0 [-18.6, 50.7] | p=0.36 |
|  |  | |
| **Both age groups**  **(ref: 2 children)** |  | |
| **>3 children** | 29.9 [11.0, 48.8] | p<0.01 |

Models adjusted for age of mother, maternal highest qualification level, living with father, season, age 4y or age 6y survey, time of week. LMVPA=light, moderate or vigorous physical activity; 95%CI=95% confidence interval.

**Table S9.5. Associations between ages of children and maternal LMVPA levels by number of children with those with missing data relating to number of younger or older children assumed to have none in that category (all days available) (n=1009)**

|  | **Beta [95%CI] for LMVPA**  **(mins per day)** | |
| --- | --- | --- |
| **1 child**  **(ref: younger children)** |  | |
| **School-aged** | -5.9 [-23.3, 11.6] | p=0.51 |
|  |  | |
| **2 children**  **(ref: younger children)** |  | |
| **School-aged** | -35.0 [-64.1, -5.9] | p=0.02 |
| **Both age groups** | -13.0 [-30.8, 4.7] | p=0.15 |
|  |  | |
| **>3 children**  **(ref: younger children)** |  | |
| **School-aged** | -82.4 [-139.5, -25.3] | p=0.01 |
| **Both age groups** | -56.7 [-102.0, -11.4] | p=0.01 |

Models adjusted for age of mother, season, age 4y or age 6y survey, time of week. LMVPA=light, moderate or vigorous physical activity; 95%CI=95% confidence interval.

**Table S9.6. Associations between number of children and maternal LMVPA levels by ages of children with those with missing data relating to number of younger or older children assumed to have none in that category (all days available) (n=913)**

|  | **Beta [95%CI] for LMVPA**  **(mins per day)** | |
| --- | --- | --- |
| **Younger children**  **(ref: 1 child)** |  | |
| **2 children** | 44.2 [19.1, 69.2] | p<0.01 |
| **>3 children** | 30.3 [-20.1, 80.6] | p=0.24 |
|  |  | |
| **School-aged**  **(ref: 1 child)** |  | |
| **2 children** | 7.3 [-16.2, 30.8] | p=0.54 |
| **>3 children** | 11.1 [-19.6, 41.9] | p=0.48 |
|  |  | |
| **Both age groups**  **(ref: 2 children)** |  | |
| **>3 children** | 22.9 [5.4, 40.4] | p=0.01 |

Models adjusted for age of mother, maternal highest qualification level, living with father, season, age 4y or age 6y survey, time of week. LMVPA=light, moderate or vigorous physical activity; 95%CI=95% confidence interval.
