# Supplementary material for: The association between number and ages of children and the physical activity of mothers: Cross-sectional analyses from the Southampton Women’s Survey
Source: PLoS One. 2022 Nov 16;17(11):e0276964. doi: 10.1371/journal.pone.0276964 (PMC9668156; doi:10.1371/journal.pone.0276964)
Supplement: Attachment — Submitted filename: recenzja PLOS ONE maj 2022.docx [file pone.0276964.s010.docx]

This is a well-conducted observational study investigating the association between number and ages of children and the physical activity of mothers based on cross sectional analyses. The authors were interested in investigating this due to previous small number of works, which were only exploratory and showed significant differences in MVPA but were not reported between mothers of children of different ages. This study is conducted appropriately with good sound research design and appropriate reason to conduct the study.

Howerver I have some minor comments:

Covariates and descriptor variables

The list of variables was established on the basis of a path analysis. Please complete the analysis with the ‘p’ values or the values of β coefficients for the relationships between covariance and dependent and independent variables. I think that for a reader with an average level of statistical knowledge, such a figure does not clearly explain how covariates were selected (description of relationships under the figure - would be a good idea).

What was the reason for the ommition of the sex of children in statistical analysis?

Statistics

220 – what was the reference group? Enter in parentheses.

Results

248-251. Fig 1. Flow chart does not apply to results. Move it to the material and methods section.

Tables

Tables do not present the exact data, please put the p values. Please take into account that most often the reader who wants to learn about the analyzes usually uses tables and figures. Hence tables must show the data clearly and precisely.

Disscusion

My comment concerns ‘possible explanations for our findings’. It was emphasized that the only significant explanation for the low physical activity of mothers of younger children and mothers of multiple children were their long, low-activity housework and childcare activities. Was it possible that other factors have played a role in maternal PA (for example: family’s income, motivation to excercise, mother’s level of education, and awareness of the importance of PA, support of other family members, BMI, and wide range of variables related to children’s: sex, sibling relations in the context of parents PA, specificity of the development of young children, their level of motor abilities…. ). Is it possible to elaborate on that?.

Appedix

I have included comments about the path analysis and tables earlier.
