# Supplementary material for: The association between number and ages of children and the physical activity of mothers: Cross-sectional analyses from the Southampton Women’s Survey
Source: PLoS One. 2022 Nov 16;17(11):e0276964. doi: 10.1371/journal.pone.0276964 (PMC9668156; doi:10.1371/journal.pone.0276964)
Supplement: Attachment — Submitted filename: PLoS One_response_to_reviewers160822.docx [file pone.0276964.s011.docx]

**Response to reviewers for ONE-D-22-10284: “The association between number and ages of children and the physical activity of mothers: cross-sectional analyses from the Southampton Women’s Survey” PLOS ONE**

We would like to thank the reviewers and Editorial Office for their helpful comments, and for providing us with the opportunity to improve the manuscript. We have responded to specific comments in the table below. We identified a minor error in the code related to MVPA analyses, but we have now corrected this and adjusted the manuscript accordingly. The related changes have been highlighted in the revised submission. They do not affect the main messages of the paper.

We look forward to your final decision,

Rachel Simpson, on behalf of all authors.

**Editorial requests**

| **Editorial request** | **Response** | **Relevant page number of main manuscript or file** |
| --- | --- | --- |
| Please ensure that your manuscript meets PLOS ONE's style requirements, including those for file naming. | We have done this. | n/a |
| Thank you for stating the following in the Competing Interests section:  [I have read the journal's policy and the authors of this manuscript have the following competing interests: CC received personal fees from Alliance for Better Bone Health, Amgen, Eli Lilly, GSK, Medtronic, Novartis, Pfizer, Roche, Servier, Takeda and UCB. KMG has received reimbursement for speaking at conferences sponsored by companies selling nutritional products, and is part of an academic consortium that has received research funding from Abbott Nutrition, Nestec, BenevolentAI Bio Ltd. and Danone. NCH has received consultancy, lecture fees and honoraria from Alliance for Better Bone Health, AMGEN, MSD, Eli Lilly, Servier, UCS, Shire, Consilient Healthcare, Kyowa Kirin and Internis Pharma. The remaining authors declare they have no competing interests.]  Please confirm that this does not alter your adherence to all PLOS ONE policies on sharing data and materials, by including the following statement: "This does not alter our adherence to  PLOS ONE policies on sharing data and materials.” (as detailed online in our guide for authors http://journals.plos.org/plosone/s/competing-interests).  If there are restrictions on sharing of data and/or materials, please state these. Please note that we cannot proceed with consideration of your article until this information has been declared.  Please include your updated Competing Interests statement in your cover letter; we will change the online submission form on your behalf. | We have added the recommended statement into the cover letter and we have changed the data availability statement to:  “Due to ethical concerns, including promises made to participants at the outset of the study that the data would not be made openly available, we are unable to share the data freely. The SWS team can provide the data on request subject to appropriate approvals. Researchers wishing to use the data would need to make a formal application to the MRC LEC Cohort Oversight Committee ([mrclec@mrc.soton.ac.uk](mailto:mrclec@mrc.soton.ac.uk)) and ensure appropriate ethical approval is in place. Subject to approval and formal agreements being signed, the data would then be provided.” | See cover letter |
| In your Data Availability statement, you have not specified where the minimal data set underlying the results described in your manuscript can be found. PLOS defines a study's minimal data set as the underlying data used to reach the conclusions drawn in the manuscript and any additional data required to replicate the reported study findings in their entirety. All PLOS journals require that the minimal data set be made fully available. For more information about our data policy, please see http://journals.plos.org/plosone/s/data-availability.  Upon re-submitting your revised manuscript, please upload your study’s minimal underlying data set as either Supporting Information files or to a stable, public repository and include the relevant URLs, DOIs, or accession numbers within your revised cover letter. For a list of acceptable repositories, please see http://journals.plos.org/plosone/s/data-availability#loc-recommended-repositories. Any potentially identifying patient information must be fully anonymized.  Important: If there are ethical or legal restrictions to sharing your data publicly, please explain these restrictions in detail. Please see our guidelines for more information on what we consider unacceptable restrictions to publicly sharing data: http://journals.plos.org/plosone/s/data-availability#loc-unacceptable-data-access-restrictions. Note that it is not acceptable for the authors to be the sole named individuals responsible for ensuring data access.  We will update your Data Availability statement to reflect the information you provide in your cover letter. | Please see competing interests response above. | Please see cover letter |
| Please include captions for your Supporting Information files at the end of your manuscript, and update any in-text citations to match accordingly. | We have done this. | Page 25 |
|  |  |  |

**Reviewer 1**

| **Comment number** | **Reviewer comment** | **Response** | **Relevant page number of main manuscript or file** |
| --- | --- | --- | --- |
| 1.1 | This manuscript proposes the need to focus on interventions and policies to increase the opportunities for higher intensity physical activity for mothers with multiple or younger children. Indeed, this manuscript provides evidence to show that specific cohorts of mothers fail to engage in sufficient physical activity and can be related to the number and age/s of their children. However, further discussion of the potential health impacts of lower volume and/or lower intensity physical activity in this population is required to highlight the significance of this research. The statistical analysis is sound; however, the authors could consider the use of an R-squared value to identify how much variation in the outcome measures are explained by physical activity and the other covariates. | Thank you very much. We have addressed the comments about lower volume and/or lower intensity physical activity under the relevant specific comments from the reviewer below (1.2-1.4; 1.12)  With regards to how much variation in the outcome measure is explained by physical activity, we would like to clarify that minutes of physical activity is the outcome measure, not the exposure and it is not therefore possible to address this. Moreover, we did not include the R-squared value for our analyses as the purpose of these analyses is to assess the association between number and ages of children and maternal physical activity rather than to predict maternal physical activity. | See below |
| 1.2 | Line 70 – Perhaps be more specific on what type (i.e. intensity) of physical activity is associated with these benefits as the discussion separates LMVPA from MVPA, with conclusions emphasising the need for performance of higher intensity activity. | Much of the evidence presented in this section is based on MET-hours per week or similar, which precludes differentiating between LPA and MVPA. However, MVPA does have additional benefits (vs. LPA), which we have detailed later on in the manuscript as requested (see page 21 of manuscript). | n/a |
| 1.3 | Line 98-99 – Please describe either here or in the methods (line 151-152) the benefits of LPA vs MVPA to 1) highlight the difference between the two intensities and 2) set the background to support the conclusion that higher intensity activity opportunities are needed for mothers. It has been identified that LPA has health benefits (line 158), but it should be explicitly discussed if the benefits are inferior or superior to MVPA or whether the volume of physical activity needs to be considered when performing LPA. | We have included additional information in relation to the difference between LPA and MVPA benefits: “MVPA was chosen as an outcome as it has been shown to have additional health benefits to LPA even when volume of physical activity energy expenditure is taken into consideration (19). However, LMVPA was also chosen as an outcome as a measure of overall physical activity, since it may be more likely that mothers of young children engage in greater LPA than MVPA, and LPA has also been shown to have health benefits (20).” | Page 7 |
| 1.4 | Line 156 – Why was LMVPA used as an outcome as opposed to just LPA? Does the addition of MVPA in the measure add any value when it is LPA that is the main concern of this measure? Could there be an interaction effect between LPA and MVPA? | LMVPA was chosen over including LPA and MVPA, as LMVPA represents total physical activity. We have now added this information in the methods: “However, LMVPA was also chosen as an outcome as a measure of overall physical activity”.  To justify this approach, we have explored correlations between the different outcome measures. We conclude that the results for LMVPA would have been very similar if LPA had been used, as the correlation between LMVPA and LPA in this dataset is r=0.97. In contrast, the correlation between LMVPA and MVPA is r=0.35. Since LMVPA and MVPA are the outcomes rather than the exposures, we did not test for an interaction. | Page 7 |
| 1.5 | Line 162 – Reference #20 specifies that 100 counts per minute is used to determine sedentary time but has been used as a threshold for LPA in the current study. Sedentary time (≤1.5 METs) is typically separate to light/low intensity physical activity (1.5-3 METs). Indeed, LMVPA is ≥100 counts, so perhaps needs further clarification of this. | The reviewer is correct in stating that 100 counts is the cut point to differentiate between time spent sedentary and time spent in light intensity activity (i.e. SED: <100cpm, LPA:≥ 100cpm). We feel this is clear in the manuscript but are happy to provide further clarification if the reviewer can describe where this might be helpful. | n/a |
| 1.6 | Line 173 – Covariates – why is BMI not included as a confounder in the model as BMI categories can influence physical activity levels? Has maternal occupation been considered? Full-time vs part-time vs unemployed, office job vs on their feet. | We used Directed Acyclic Graphs to identify which covariates were needed in each model (DAGs presented in S2 Appendix and S3 Appendix). This provides a robust, objective method to assess which variables are appropriate to adjust for in an analysis (Tennant PWG, Murray EJ, Arnold KF, Berrie L, Fox MP, Gadd SC et al. (2021) Use of directed acyclic graphs (DAGs) to identify confounders in applied health research: review and recommendations. Int J Epidemiol 50, 620-32; Rohrer JM (2018) Thinking Clearly About Correlations and Causation: Graphical Causal Models for Observational Data. Adv Methods Pract Psychol Sci 1, 27-42). Maternal BMI and maternal employment were included in the DAG, but were not found to be confounders of potential causal relationships between exposures and outcomes so were not included in the models. We have now clarified which variables were included in the DAG in the main text as well as in the diagrams themselves: “Variables assessed in the DAGs were living with the father of the index child, maternal education, maternal age, maternal body mass index (BMI), maternal employment and pre-school attendance.” | Page 9 |
| 1.7 | Line 252 – Please specify sample sizes for age 4y and 6y in each level of the flow diagram chart. | We have added in ** and *** to the relevant parts of the flow diagram with the following caption: “*n=621 for age 4y and n=608 for age 6y. **n=607 for age 4y and n=592 for age 6y. ***n=457 for age 4y and n=525 for age 6y.” | Page 11 and Fig 1. |
| 1.8 | Line 299 – For consistency, consider use of just tables or graphs for presenting associations between number and ages of children and MVPA or LMVPA (i.e. table 4, figure 2 and 3). | If the editor agrees, we would prefer to keep the graphs for the stratified analyses and the tables for the unstratified analyses. We believe the graphs convey the complexity of the stratified analyses more clearly. | n/a |
| 1.9 | Line 373 – The group that performed less MVPA still met the physical activity guidelines, is this comparable to the current study? It is worth addressing whether the groups stratified by age/number of children, when describing the percentage change in physical activity, met the physical activity guidelines. | The results of this previous study are not comparable to the current study. We have added in the statement, “However, unlike in our study in which the average amount of MVPA was below the recommended guidelines regardless of child age (see Tables 2 and 3), the average amount of MVPA for all mothers in Candelaria et al. 2012 was above the recommended guidelines (10).” We state the median daily MVPA for mothers by ages of children in Tables 2 and 3. | Page 18, Tables 2 and 3 |
| 1.10 | Line 399 – Consider highlighting the limitations of self-report data vs accelerometer assessed data. | We state “the use of accelerometer assessment of physical activity is a key strength, important to ensure that sporadic and spontaneous movement was captured, which is likely to be common amongst mothers of younger children and might be easily missed in self-reported physical activity” and “We also do not know the types of physical activity the women engaged in or their locations whilst wearing the accelerometer” (Page 20). We have now added:  “Studies assessing physical activity through a combination of device-assessment and self-report would also be helpful to provide information on both amount and type of physical activity engaged in by mothers.” (Page 21) | Pages 20-21 |
| 1.11 | Line 425 – One potential limitation to this study is the representativeness of this study cohort to the current population of mothers, given the changes in physical activity culture and opportunities in 2022 vs 2000-2012. Please address this. | Few studies have assessed physical activity of parents using accelerometers so the SWS data provides a rare opportunity to conduct these analyses. As regards dates, the need to increase physical activity levels among adults has remained constant. We have addressed this comment in the following addition:  “The physical activity data is from 2006 to 2012 but is still highly relevant considering that physical activity levels amongst adults in high-income western countries have since continued to decline (34).” | Page 20 |
| 1.12 | Line 448-449 – Please discuss in further detail why women who already undertake large amounts of LPA (likely involving playing with and picking up young children) need to engage in additional MVPA? Is this for health benefits that are only related to MVPA rather than LPA? Is LPA of mothers different to LPA of non-parents in terms of intensity and duration? Could the volume of LPA be enough to see other health benefits while also being more achievable and feasible for mothers? | There are indeed additional health benefits from engaging in MVPA. We have now added:  “LPA does provide health benefits, but in a study examining the association between device-assessed physical activity and mortality rates, greater reductions were achieved amongst those reaching a set physical activity energy expenditure through higher intensity physical activity than through more time spent in lower intensity activity (19).” | Page 21 |

**Reviewer 2**

| **Comment number** | **Reviewer comment** | **Response** | **Relevant page number of main manuscript or file** |
| --- | --- | --- | --- |
| 2.1 | This is a well-conducted observational study investigating the association between number and ages of children and the physical activity of mothers based on cross sectional analyses. The authors were interested in investigating this due to previous small number of works, which were only exploratory and showed significant differences in MVPA but were not reported between mothers of children of different ages. This study is conducted appropriately with good sound research design and appropriate reason to conduct the study. | Thank you very much | n/a |
| 2.2 | Covariates and descriptor variables The list of variables was established on the basis of a path analysis. Please complete the analysis with the ‘p’ values or the values of β coefficients for the relationships between covariance and dependent and independent variables. I think that for a reader with an average level of statistical knowledge, such a figure does not clearly explain how covariates were selected (description of relationships under the figure - would be a good idea). | Covariates were chosen using Directed Acyclic Graphs, which are theoretical rather than analytical tools now recognised to best inform the choice of covariates to include in a model. Direct Acyclic Graphs are therefore distinct from a path analysis and so there are no p values or beta coefficients to display. | n/a |
| 2.3 | What was the reason for the omission of the sex of children in statistical analysis? | Data on sex of the children were only available for the index child, and not for their siblings. We have added this in as a limitation:  “We lacked data on sex of children other than the index child so this variable was not included in the models.” | Page 20 |
| 2.4 | 220 – what was the reference group? Enter in parentheses. | There are various reference groups depending on the analysis (see results tables and figures for the reference groups). We have added in “compared with the reference group for each analysis” to clarify this. | Page 10 |
| 2.5 | 248-251. Fig 1. Flow chart does not apply to results. Move it to the material and methods section. | This has been moved as suggested. | Page 11 |
| 2.6 | Tables do not present the exact data, please put the p values. Please take into account that most often the reader who wants to learn about the analyzes usually uses tables and figures. Hence tables must show the data clearly and precisely. | We appreciate this suggestion, but prefer to include 95% confidence intervals rather than p-values as they provide evidence of the magnitude and range of effect sizes rather than just statistical significance (Please see Karpen et al. 2017 - <https://www.ncbi.nlm.nih.gov/pmc/articles/PMC5738950/>; Amrhein et al. 2019 - https://www.nature.com/articles/d41586-019-00857-9) | n/a |
| 2.7 | My comment concerns ‘possible explanations for our findings’. It was emphasized that the only significant explanation for the low physical activity of mothers of younger children and mothers of multiple children were their long, low-activity housework and childcare activities. Was it possible that other factors have played a role in maternal PA (for example: family’s income, motivation to exercise, mother’s level of education, and awareness of the importance of PA, support of other family members, BMI, and wide range of variables related to children’s: sex, sibling relations in the context of parents PA, specificity of the development of young children, their level of motor abilities…. ). Is it possible to elaborate on that?. | We agree with the reviewer that many of these other factors could affect the physical activity of mothers, but in most cases, this would be unlikely to vary by ages or number of children. Therefore, we have not made any related changes to the manuscript | n/a |
|  |  |  |  |

**Reviewer 3**

| **Comment number** | **Reviewer comment** | **Response** | **Relevant page number of main manuscript or file** |
| --- | --- | --- | --- |
| 3.1 | Thank you for giving me the opportunity to review this manuscript. The approach with the use of an accelerometer in the assessment of PA is rare so the material becomes more interesting. But it needs more clarification whether this double publication or not because part of this result like ''Women with any school-aged children engaged in more MVPA than those with only <4y e.g.% difference in minutes of MVPA [95%CI]: 42.2% [18.7-70.4] for mothers with only school aged vs only <4y). Mothers with multiple children did less MVPA than those with 1 child (e.g.51 13.9% [1.0-25.2] less MVPA for those with 2 children almost similar to the result published by the same authors titled as ''Cross-sectional associations between number and ages of children and maternal physical activity ''Published: 20 October 2021 on European Journal of Public Health, Volume 31, Issue Supplement_3, October 2021, ckab164.789, https://doi.org/10.1093/eurpub/ckab164.789. | Thank you very much. This is not a dual publication. The abstract referred to is from the European Public Health Conference 2021 which was mentioned in our cover letter to the editor on submission. | n/a |
| 3.2 | The abstract contains some short forms of words that were not written in the full form first. | We have changed y to y(ears) and CI to confidence interval. | Page 2 |
| 3.3 | Introduction: In the first paragraph ref.1-3 all discuss the benefits of PA but ref 2 a number of times. It is better if ideas merged together and are referenced at last. | We appreciate this suggestion but have not changed this as we feel that it is important for the reader to be able to differentiate the point that each reference relates to, instead of having all references grouped together at the end of a sentence. | Page 4 |
| 3.4 | I suggest supporting your conclusion part in the main document at the end with your finding than mentioning your title | Thank you for this suggestion. We have changed the conclusion to “Policies and interventions are needed to encourage mothers with younger or multiple children to engage in greater MVPA, ensuring they benefit from health gains associated with higher intensity activity.” | Page 21 |
|  |  |  |  |

**Reviewer 4**

| **Comment number** | **Reviewer comment** | **Response** | **Relevant page number of main manuscript or file** |
| --- | --- | --- | --- |
| 4.1 | The authors have taken a very important and novel aim to investigate the associations between ages and number of children and device-measured maternal physical activity (PA). This study also aimed at identifying the subgroups of mothers who are more at risk of insufficient PA than others. This work has a great practical value. The authors made a huge research effort to collect data over so many years. | Thank you very much | n/a |
| 4.2 | Please be consistent with specifying the groups of children. In the line 42 you have: “the index child was aged 4y or 6y” and in lines 108-110: the aim of this cross-sectional study is to investigate the association between number and ages of children, categorized as >5y (hereafter referred to as school aged) and ≤ 4 y…. Even though the authors explains the categories of mothers in the Table 1, it is very confusing for the reader to understand. | We apologise for this confusion. The first relates to the age of the index child at the time of the survey, the second are age groups as used in the analyses. For clarity, we have removed the reference to the age of the index child in the abstract and replaced it with “at two different timepoints”. | Page 2 |
| 4.3 | I think it should be stated already in the abstract that the surveys were performed in two time points. | See previous comment (4.2) | Page 2 |
| 4.4 | You surveyed the women between March 2006 and June 2009 and between March 2007 and August 2012. Were there women in this group who participated in the survey twice? | Yes – on page 6 we mention that “Valid accelerometer data were available for 621 and 608 women at the 4y and 6y waves respectively (total n=1034; n=195 women with both timepoints).” Repeat assessments were accounted for in the multi-level analyses. | Page 6 |
| 4.5 | Lines 182-183: Please explain “maternal highest qualification level from pre-pregnancy data (6 categories ranging from none to degree) – what are these 6 categories? | We have now included this: “none; certificate of secondary education; O-levels, A-levels; higher national diploma; degree” | Page 9 |
| 4.6 | I totally agree with the statement (430 – 433) that a key strength of this work was the use of accelerometers to assess PA, important to ensure that sporadic and spontaneous movement was captured, which is likely to be common amongst mothers of younger children and might be easily missed in self-reported PA. It would valuable to translate obtained outcomes to practical recommendations on how to increase PA levels in mothers from specific subgroups, e.g how to increase the MVPA of mothers with younger children. For future studies it would be also valuable to collect data (based on questionnaires) which kind of PA the mothers were involved in, e.g. transportation, household activities, supervised sessions, etc. It could be helpful to develop proper strategies to increase the PA level, especially in mothers who are more at risk of insufficient PA than others. | We agree that all of these areas would be key topics for future research. We do not have data on domain of physical activity, but we agree that a combination of self-reported physical activity and objectively measured physical activity would be very helpful. We have added the following sentence: “Studies assessing physical activity through a combination of device-assessment and self-report would also be helpful to provide information on both amount and type of physical activity engaged in by mothers.” | Page 21 |
| 4.7 | Please be consistent throughout the entire text to use the term Physical activity or the “PA” abbreviation. | We have checked the manuscript; this is now consistent throughout the manuscript text, but we have retained PA in the abstract for brevity. | n/a |
| 4.8 | Minor comment: I suggest to change the reference for an updated version of the WHO guidelines (WHO, 2020). WHO. (2020). WHO guidelines on physical activity and sedentary behaviour. Geneva: World Health Organization. In (Vol. 57). Geneva: World Health Organization. | We have changed the 2018 WHO reference to the 2020 equivalent. | See reference list |
