# Supplementary material for: The association between number and ages of children and the physical activity of mothers: Cross-sectional analyses from the Southampton Women’s Survey
Source: PLoS One. 2022 Nov 16;17(11):e0276964. doi: 10.1371/journal.pone.0276964 (PMC9668156; doi:10.1371/journal.pone.0276964)
Supplement: Attachment — Submitted filename: PLoS One_response_to_reviewers160922.docx [file pone.0276964.s012.docx]

**Response to reviewers for ONE-D-22-10284: “The association between number and ages of children and the physical activity of mothers: cross-sectional analyses from the Southampton Women’s Survey” PLOS ONE**

We would like to thank the reviewers and Editorial Office for their helpful comments, and for providing us with the opportunity to improve the manuscript. We have responded to specific comments in the table below.

We look forward to your final decision,

Rachel Simpson, on behalf of all authors.

**Editorial requests**

| **Editorial request** | **Response** | **Relevant page number of main manuscript or file** |
| --- | --- | --- |
| Please review your reference list to ensure that it is complete and correct. If you have cited papers that have been retracted, please include the rationale for doing so in the manuscript text, or remove these references and replace them with relevant current references. Any changes to the reference list should be mentioned in the rebuttal letter that accompanies your revised manuscript. If you need to cite a retracted article, indicate the article’s retracted status in the References list and also include a citation and full reference for the retraction notice. | We have reviewed the reference list and it is complete and correct. We have checked and there is no evidence that any of the papers have been retracted. The World Health Organisation now have a different webpage with information relating to classification of people by BMI. However, we have kept the original one (reference 24) with the date which we accessed to accurately reference the methods and terminology used in our paper. | Pages 23-24 |

**Reviewer 2**

| **Comment number** | **Reviewer comment** | **Response** | **Relevant page number of main manuscript or file** |
| --- | --- | --- | --- |
| 2.1 | Thank you for replying to the review. I read them in detail. I agree with most. However, section 2.6 requires additional solutions. I agree that there are different approaches to statistical analyzes, nevertheless, the presented data should give us a full answer to research questions. If the authors do not want to present the p-value, please also provide the effect size with a 95% CI. (or alternatively p-value). One of the two solutions will be accepted. I read the articles cited with interest, as well as others on p-value. The value of a 95% CI does not tell us about an estimate, just as the p-value does not give us an answer about the research hypothesis. | We would like to clarify that we did present information on the magnitude of the effect by providing the estimates in all tables and in the text. We have retained this, but at the request of the reviewer, we now also provide p-values in Table 4 and the tables in S5, S8 and S9 Appendices. | See Table 4, Page 15 and S5, S8 and S9 Appendices |

**Reviewer 3**

| **Comment number** | **Reviewer comment** | **Response** | **Relevant page number of main manuscript or file** |
| --- | --- | --- | --- |
| 3.1 | Thank you authors for your interesting work. All my comments have been adequately addressed. Now the paper can be acceptable for publication. | Thank you very much | n/a |

**Reviewer 4**

| **Comment number** | **Reviewer comment** | **Response** | **Relevant page number of main manuscript or file** |
| --- | --- | --- | --- |
| 4.1 | The authors have taken a very important and novel aim to investigate the associations between ages and number of children and device-measured maternal physical activity. This work has a great practical value and certainly should be published. | Thank you very much. | n/a |
