# Supplementary material for: The association between number and ages of children and the physical activity of mothers: Cross-sectional analyses from the Southampton Women’s Survey
Source: PLoS One. 2022 Nov 16;17(11):e0276964. doi: 10.1371/journal.pone.0276964 (PMC9668156; doi:10.1371/journal.pone.0276964)
Supplement: Attachment — Submitted filename: response_plos_one.docx [file pone.0276964.s013.docx]

Dear Autors,

thank you for replying to my review. I read them in detail. I agree with all comments. I believe, your article meets all the criteria and should be appear in the scientific journal: PLOS ONE
